# Recent Advances and Challenges in Ti-Based Oxide Anodes for Superior Potassium Storage

**DOI:** 10.3390/nano13182539

**Published:** 2023-09-11

**Authors:** Qinglin Deng, Yang Zhao, Xuhui Zhu, Kaishuai Yang, Mai Li

**Affiliations:** 1 School of Physics and Materials Science, Guangzhou University, Guangzhou 510006, China; zy843795537@163.com (Y.Z.); zxh2674091377@163.com (X.Z.); 2 Research Center for Advanced Information Materials (CAIM), Huangpu Research & Graduate School of Guangzhou University, Guangzhou 510555, China; 3 School of Electronic and Information Engineering, Changshu Institute of Technology, Suzhou 215000, China; 4 College of Science, Donghua University, Shanghai 201620, China

**Keywords:** Ti-based oxides, anodes, potassium-ion batteries, intercalation type, energy storage

## Abstract

Developing high-performance anodes is one of the most effective ways to improve the energy storage performances of potassium-ion batteries (PIBs). Among them, Ti-based oxides, including TiO_2_, K_2_Ti_6_O_13_, K_2_Ti_4_O_9_, K_2_Ti_8_O_17_, Li_4_Ti_5_O_12_, etc., as the intrinsic structural advantages, are of great interest for applications in PIBs. Despite numerous merits of Ti-based oxide anodes, such as fantastic chemical and thermal stability, a rich reserve of raw materials, non-toxic and environmentally friendly properties, etc., their poor electrical conductivity limits the energy storage applications in PIBs, which is the key challenge for these anodes. Although various modification projects are effectively used to improve their energy storage performances, there are still some related issues and problems that need to be addressed and solved. This review provides a comprehensive summary on the latest research progress of Ti-based oxide anodes for the application in PIBs. Besides the major impactful work and various performance improvement strategies, such as structural regulation, carbon modification, element doping, etc., some promising research directions, including effects of electrolytes and binders, MXene-derived TiO_2_-based anodes and application as a modifier, are outlined in this review. In addition, noteworthy research perspectives and future development challenges for Ti-based oxide anodes in PIBs are also proposed.

## 1. Introduction

### 1.1. Advantages of Potassium-Ion Batteries

With the widespread use and rise of portable electronic products and new energy vehicles, the demand for energy storage secondary batteries that rely on their operation is increasing [1,2]. Lithium-ion batteries (LIBs) are currently some of the most successful and widely used commercial secondary batteries [3]. Based on the unique physical and chemical properties of lithium, they bring lots of energy storage advantages, such as high energy density, high working voltage, long cycle life and so on [4]. However, the reserves of lithium in the crust are only 0.0017%, and the current LIBs have an immature recycling mechanism, which has increased their production cost [5,6]. In the long run, this will inevitably affect their supply and demand. In order to compensate for and solve the cost bottleneck of LIBs, researchers are currently shifting their focus to cheaper sodium-ion batteries (SIBs) and potassium-ion batteries (PIBs) [7,8]. The reserves of sodium/potassium in the crust are very abundant, with 2.3% of sodium and 1.5% of potassium in the crust, which are rarely limited by geographical conditions [9,10]. However, as we know, it is difficult for Na-ions to react with commercial graphite. The resulting compounds are also very unstable, which greatly affects the further application of SIBs [11]. Fortunately, potassium can form stable KC_8_ intercalation compounds with graphite [12]. In addition, compared with SIBs, PIBs have the following unique advantages: 1. The standard electrode potential of potassium is −2.93 V (vs. SHE), which is close to that of lithium (−3.04 V vs. SHE) and higher than that of sodium (−2.71 V vs. SHE), indicating that PIBs may have higher energy density. 2. K-ions have lower Lewis acidity and a smaller solvation ionic radius, which makes K-ions have higher ionic conductivity than sodium ions in water or non-aqueous electrolytes. 3. Similar to sodium, potassium also does not form alloys with Al; therefore, Al foil can also be used as a current collector. 4. KPF_6_ electrolyte is cheaper than NaPF_6_ in the same quality and has better chemical stability [8,13,14,15]. Therefore, the research on PIBs has important research significance and broad application prospects.

### 1.2. Anodes of PIBs

In recent years, PIBs have received widespread attention from researchers, and the published papers have also shown an upward trend [13]. The working principles of PIBs are very similar to LIBs and SIBs. They are also based on the “rocking chair” working mechanism. The charging and discharging are realized by the reversible insertion/extraction of K-ions between positive and negative electrodes [16]. In terms of the structure and composition of PIBs, they are essentially the same as the LIBs and SIBs, which are mainly composed of anodes, cathodes, separations, electrolytes and outer shells.

According to different potassium storage mechanisms, the anodes of PIBs can be divided into three types: alloy-type, conversion-type and intercalation-type [17,18,19,20]. Alloy-type anode materials can undergo multi-electron alloying reactions with potassium, thus possessing high theoretical specific capacity. However, the alloying process will be accompanied by huge volume expansion, ultimately leading to the gradual pulverization, peeling and invalidity of the active materials. At present, alloy-type anode materials mainly include Sn-based, Bi-based, Sb-based, etc. [21,22,23]. Conversion-type anode materials are based on the conversion reaction mechanism with high theoretical specific capacity and redox reversibility. They usually have a lower volume expansion rate than alloy-type anodes during the charging and discharging process. The conversion-type anode materials mainly include some metal oxides, sulfides, selenides, etc. [18,19,24,25]. However, there are also many shortcomings that need to be urgently addressed for the application of conversion-type anodes in PIBs, such as low Coulombic efficiency at the first cycle, poor rate and cycle stability performances, etc.

Unlike alloy-type and conversion-type anodes, the theoretical specific capacity of intercalation-type anodes is usually lower [26]. As for PIBs, owing to the intercalation mechanism in the charging and discharging process, intercalation-type anodes can suffer smaller volume expansion effects during the insertion/extraction process of K-ions, exhibiting better rate and cycling stability performances. The intercalation-type anode of PIBs mainly includes carbon materials and some oxides [20,27,28,29]. According to different graphitization degrees, carbon materials can be generally divided into graphite, soft carbon, hard carbon and graphene. Lots of research has shown that K-ions can be embedded into graphite, generating stable KC_8_ compounds [30]. Non-graphitized hard carbon and soft carbon have a wide interlayer spacing and a large degree of disorder, which is very conducive to the insertion/extraction of K-ions [27]. In addition, typical intercalation-type oxides include TiO_2_, Nb_2_O_5_ and layered K_2_Ti_4_O_9_, etc. [16,20,31]. They usually have good chemical and thermal stability, as well as environmental friendliness, and are currently the popular anode materials for PIBs.

### 1.3. Ti-Based Oxide Anodes

As we can see, based on different potassium storage mechanisms, these three types of anodes present different energy storage advantages and disadvantages in PIBs. Intercalation-type anodes have better rates and cycling performances, showing enormous application potential in large-scale energy storage and smart grids. Among them, Ti-based oxide anodes are gaining more attention owing to their low cost, rich reserves of Ti elements, non-toxic and environmentally friendly properties and high stability [32,33]. The Ti element has rich redox chemistry of Ti^4+^/Ti^3+^, which is beneficial for electrochemical storage applications. Ti-based oxides, such as TiO_2_, Li_4_Ti_5_O_12_, Na_2_Ti_3_O_7_, etc., have been widely studied in LIBs and SIBs [34,35,36]. Based on their inherent structural advantages, they theoretically have great potential for application in PIBs. However, compared to their applications in LIBs and SIBs, there is relatively little research on their application in PIBs.

**Figure 1:**
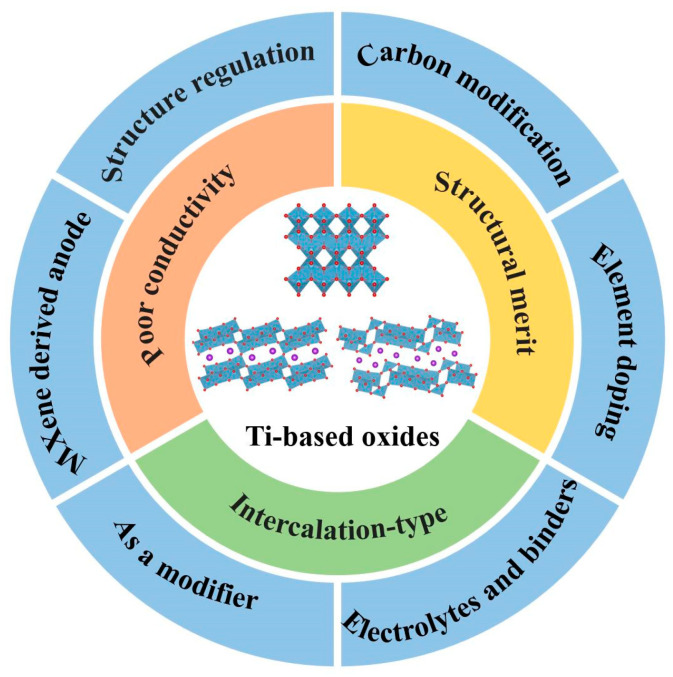
Energy storage characteristics and research directions of Ti-based oxide anodes in PIBs.

As far as we know, there are few comprehensive reviews on Ti-based oxide anodes focusing on applications in PIBs. Compared with previous similar work, the application in LIBs and SIBs is their research emphasis [32,33,37]. In this work, we systematically review Ti-based oxide anodes, including TiO_2_ and the A-Ti-O (A = Li, Na, K) family, etc., for application in PIBs. Figure 1 shows the energy storage characteristics and research directions of Ti-based oxide anodes in PIBs. Based on the latest published literature, some related important work is discussed in detail in this review. Besides some important classification viewpoints, such as the potassium storage mechanism, structural regulation, carbon modification and element doping, some other novel perspectives, including the effects of electrolytes and binders, MXene-derived TiO_2_-based anodes and the application as a modifier, are outlined in this review. In addition, we share some noteworthy perspectives on future challenges for Ti-based oxide anodes in potassium storage application.

## 2. TiO_2_

### 2.1. Research on Potassium Storage Mechanism

**Figure 2:**
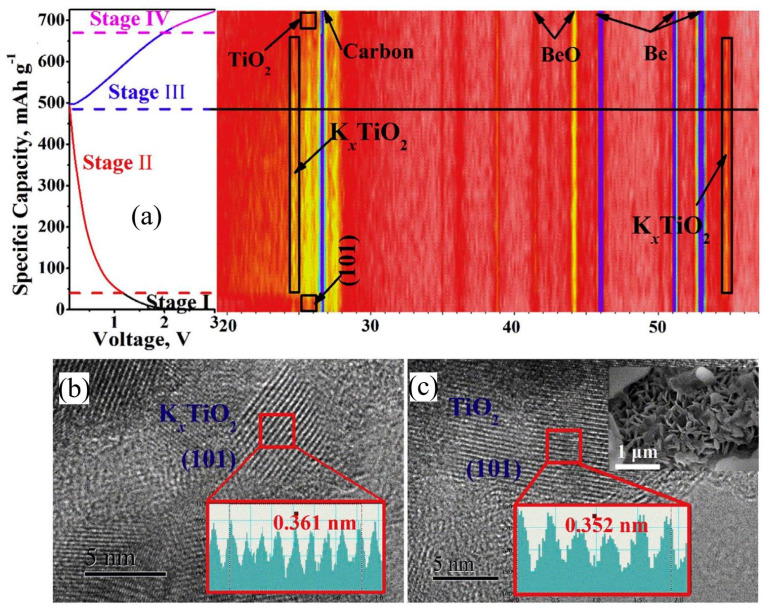
(**a**) In situ XRD patterns of HeTiO_2_eC MTs anode operated at different charge states at 100 mA g^−1^. HR-TEM of HeTiO_2_eC MTs at discharge state of 0.01 V (**b**) and at charge state of 3.0 V (**c**). Reproduced with permission from Ref. [40]. Copyright 2019 Elsevier.

As a typical representative of Ti-based oxides, TiO_2_ has been widely used as the anode for application in energy storage batteries, showing numerous advantages. It has made important research progress in LIBs and SIBs [38,39]. Based on its inherent structural advantages, TiO_2_ also exhibits potential application in PIBs. In order to further explore and optimize the potassium storage characteristics of TiO_2_, it is necessary to study its K-ion insertion/extraction mechanism. Yang et al. [40] prepared a hierarchical HeTiO_2_eC micro-tube (MTs) heterostructure, which shows enhanced potassium storage performances. They also investigated its K-ion insertion/extraction mechanism using the in situ XRD technique. As shown in Figure 2a, the relevant reaction mechanism of the HeTiO_2_eC electrode can be revealed in four stages. In stage (II), two new diffraction peaks can be associated with K*_x_*TiO_2_, indicating the existence of intercalation behavior. In combination with electrochemical testing and in situ XRD results, K-ion insertion/extraction into/from TiO_2_ can be expressed as

K-ions insertion: TiO_2_ + *x*K^+^ + *x*e^−^ → K*_x_*TiO_2_ (1)

K-ions extraction: K*_x_*TiO_2_ → TiO_2_ + *x*K^+^ + *x*e^−^ (2)

Among them, *x* represents the mole fraction of the inserted K-ions. This K-ion reaction mechanism is consistent with the observed phenomenon of the HRTEM experiment, which was conducted at a discharge state of 0.01 V (shown in Figure 2b) and a fully charged state of 3.0 V (shown in Figure 2c). The changes in latticed-spacing value indicate that the insertion of K-ions into TiO_2_ leads to slight volume expansion [40]. Moreover, Sun et al. [41] also certified the consistent K-ion storage mechanism of TiO_2_-based electrodes using in situ TEM and operando XRD techniques. In addition, Dambournet et al. [42] studied the K-ion intercalation behavior in lepidocrocite-type layered TiO_2_ structures using first-principle electronic structure calculations. Their research results indicate that Ti^4+^ vacancies are unable to accommodate K-ions without significant distortion.

### 2.2. Structural Regulation, Carbon Modification and Element Doping

**Figure 3:**
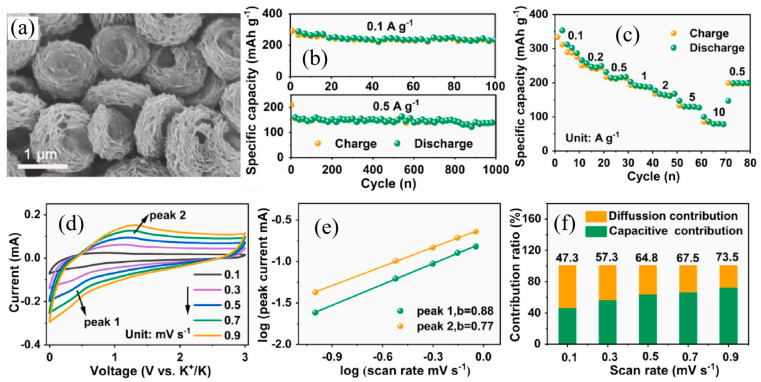
(**a**) FESEM images of TiO_2_/NC-HN. Cycling stability (**b**) and rate performance (**c**) of TiO_2_/NC-HN electrode. (**d**) CV curves at different scan rates; (**e**) the b-values determined by scan rates and peak currents; (**f**) normalized ratio of capacitive and diffusion at different scan rates, for TiO_2_/NC-HN electrode. Reproduced with permission from Ref. [45]. Copyright 2020 Elsevier.

Like other similar oxide anodes, the large bandgap of TiO_2_ indicates its poor electrical conductivity, which seriously restricts the release of its potassium storage performances [40]. This is also the most critical problem that needs to be solved currently if significant development in PIBs is to be achieved. Researchers have developed numerous modification and optimization methods to improve their potassium storage performances. An excellent anode should have significant improvements in specific capacity, rate and cycling performances. There are many modification and optimization methods for TiO_2_. Specifically, the effective combination of structural optimization and carbon modification has been certified to markedly improve the potassium storage performances of TiO_2_. Effective carbon coating modification has been proven to significantly improve the potassium storage performance of electrode materials [43,44]. As shown in Figure 3a, Zhang et al. [45] designed an effective nanocomposite structure of nest-like TiO_2_-nitrogen-doped carbon (TiO_2_/NC-HN), which shows excellent potassium storage performances. Owing to the synergistic effects of hierarchical hybrid nanostructures, nitrogen-doped carbon and the possible oxygen vacancy in TiO_2_, excellent cycling performance can be observed at 0.1 and 0.5 A g^−1^ (shown in Figure 3b). This composite electrode also delivers a high specific capacity of 277.2, 244.7, 210.2, 185.5, 165.7, 126.9 and 79.1 mA h g^−1^ at the current density of 0.1, 0.2, 0.5, 1.0, 2.0, 5.0 and 10.0 A g^−1^, respectively (shown in Figure 3c). It also maintains a high capacity retention of 85.7% after 1500 cycles at 2 A g^−1^. In addition, the CV results tested at different scan rates indicate the capacitive-diffusive hybrid charge storage mechanisms for the TiO_2_/NC-HN electrode (shown in Figure 3d–f) [45].

As we know, graphene is often used as a modifier to improve the electrical conductivity of active materials [46]. Graphene was grown on the surface of TiO_2_ nanotubes (G-TiO_2_ NTs) by Sun et al. [41] using the chemical vapor deposition technique. The introduction of graphene coatings can not only enhance its conductivity but can also alleviate the volume expansion during the potassium storage process. As a result, it shows a superior rate performance with a high specific capacity of 271.6, 258.7, 217.3, 189.3, 166.8, 133.4 and 129.2 mA h g^−1^ at 0.05, 0.1, 0.2, 0.5, 1, 2 and 5 A g^−1^, respectively. It also exhibits a high capacity retention of 84.1% after 400 cycles at 0.1 A g^−1^ [41]. In addition, other structure- and carbon-modified TiO_2_-based materials, such as carbon-coated flower-like TiO_2_ nanosphere [47], sandwich-like structured TiO_2_/graphene composite [48], hierarchical HeTiO_2_eC micro-tubes [40], TiO_2_ nanoparticle supported N-rich graphitic porous carbon [49] and TiO_2_-coated polyaniline intercalated layered titanate [50], show superior potassium storage performances.

**Figure 4:**
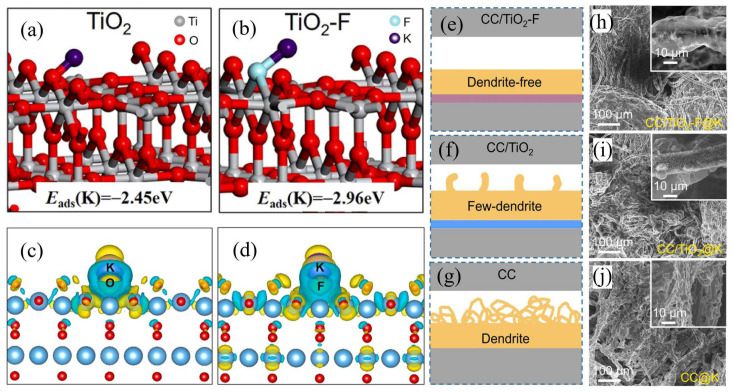
Detailed model construction of K on rutile TiO_2_ (**a**) and F-doped TiO_2_ (**b**) (110) face. Calculated spin-polarized charge densities of TiO_2_ (**c**) and F-TiO_2_ (**d**) upon K adsorption. Schematic illustration for CC/TiO_2_-F (**e**), CC/TiO_2_ (**f**) and bare CC (**g**) of K plating. SEM images of CC/TiO_2_-F (**h**), CC/TiO_2_ (**i**) and CC (**j**) after K plating. Reproduced with permission from Ref. [56]. Copyright 2021 Elsevier.

Besides structural regulation and carbon modification, element doping is also commonly used to improve the energy storage performances of anode materials [51]. As for TiO_2_, doping with some elements such as Nb, N and S could effectively modify its electronic structure and then further enhance its energy storage performances [52,53,54]. Cao et al. [55] prepared Ta-doped TiO_2_/C nanofibers using an electrospinning strategy. The theoretical calculation results indicate that the K diffusion barrier in the TiO_2_ electrode can be lowered by Ta doping. Owing to the improved electrical conductivity and phase transformation, etc., this composite electrode exhibits a high specific capacity of 148.8 mA h g^−1^ in PIBs at the current density of 2 A g^−1^ after 800 cycles [55]. In addition, as shown in Figure 4, Shao et al. [56] proposed a strategy for inducing uniform nucleation of K through TiO_2_ nanorod arrays by doping with F heteroatoms. The theoretical calculation results (shown in Figure 4a–d) indicate that a denser electron cloud can be accumulated at the interface between TiO_2_-F and K. As shown in Figure 4e–j, compared with the carbon cloth (CC), CC/TiO_2_ and CC/TiO_2_-F electrodes during the plating process, CC/TiO_2_-F shows a smoother K deposition morphology; however, K dendrites are observed on bare CC and CC/TiO_2_ electrodes. K-ions can strongly adhere to F heteroatoms with electronegativity, resulting in enhanced nucleophilicity [56]. Such an F element doping strategy can help us develop dendrite-free metal anodes for promoting the research and application in PIBs.

### 2.3. MXene-Derived TiO_2_-Based Anodes

MXenes, as a class of two-dimensional (2D) transition metal carbides and nitrides, have received a lot of attention from researchers [57,58,59]. They contain covalent bonds, metal and ionic bonds and adjustable functional groups, which can provide many advantages for preparing their composites and derivatives. Owing to the abundant Ti sources of Mxene, its derived TiO_2_-based sample can be easily obtained using the heat treatment method. He et al. [60] prepared layered Ti_3_C_2_/TiO_2_/rGO (reduced graphene oxide) nanosheets using Mxene Ti_3_C_2_-generated TiO_2_ nanoparticles and rGO sheets. Owing to the structural advantages of Mxene and rGO, this composite electrode shows superior potassium storage performances (349.2 mA h g^−1^ after 200 cycles at 0.1 A g^−1^, 229.3 mA h g^−1^ after 500 cycles of 0.5 A g^−1^). Similarly, Cao et al. [61] used Ti_2_C MXene-derived TiO_2_ nanoparticles and rGO nanosheets to synthesize a 2D TiO_2_/rGO composite, which shows enhanced potassium storage performances. Mxene can not only utilize its Ti atoms to prepare TiO_2_ but also uses its structural advantages to construct heterostructures. Sun et al. [62] designed a novel Mxene-derived TiSe_2_/TiO_2_/C heterostructure. Ti atoms in Mxene can be converted into TiSe_2_/TiO_2_ by a heat treatment process. The presence of a built-in electric field in this heterogeneous interface is beneficial for the electrochemical potassium storage process. As for the application in PIBs, it displays a specific capacity of 121 mA h g^−1^ at 0.1 A g^−1^ after 800 cycles.

**Figure 5:**
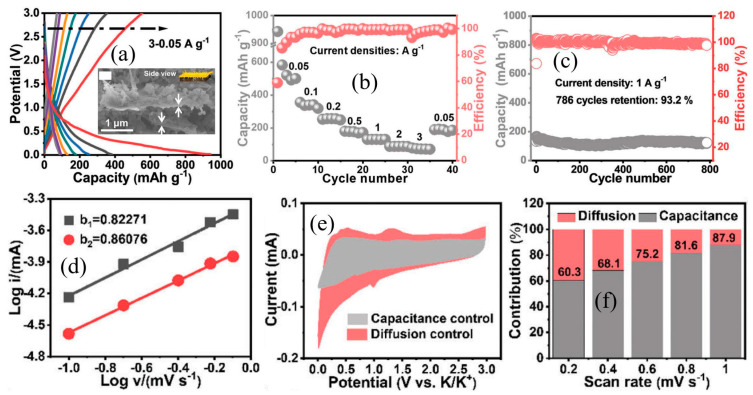
Electrochemical test of MOF-NOC@L-TiO_2_ electrode for PIBs: (**a**) Charge–discharge curves, (**b**) rate performance, (**c**) cycling stability, (**d**) the b-values determined by scan rates and peak currents, (**e**) CV curve at the scan rate of 0.2 mV s^−1^ and (**f**) normalized ratio of capacitive and diffusion at different scan rates. The inset of (**a**) is side views for SEM of MOF-NOC@L-TiO_2_. Reproduced with permission from Ref. [63]. Copyright 2023 Wiley.

As we know, Mxene-derived layered titania (L-TiO_2_) has many advantages for application in PIBs [63]. However, its unstable layered structure and poor conductivity restrict its potassium storage performances. As shown in Figure 5, inspired by the “sand-fixation model” in nature, Sun et al. [63] developed a novel MOF-NOC (interconnected N/O-doped carbon)@L-TiO_2_ anode. The transformed MOF-NOC plays a positive role in suppressing the pulverization of L-TiO_2_ and improving the stability and conductivity of composite electrodes. As for the application in PIBs, it shows a high specific capacity of 555, 355, 253, 179, 130, 87 and 73 mA h g^−1^ at 0.05, 0.1, 0.2, 0.5, 1, 2 and 3 A g^−1^, respectively (shown in Figure 5a,b). It also has a high capacity retention of 93.2% after 786 cycles at 1 A g^−1^ (shown in Figure 5c). In addition, the CV test results indicate the main kinetics of pseudocapacitive and double-layer capacitive characteristics for the MOF-NOC@L-TiO_2_ electrode (shown in Figure 5d–f) [63].

### 2.4. Effects of Electrolytes and Binders

As we know, modifying and optimizing all components of PIBs, including cathodes, anodes, electrolytes and binders, etc., can effectively improve their energy storage performances [12,64,65,66]. However, most researchers currently focus on improving the potassium storage performance of TiO_2_ through structural optimization, carbon modification and element doping, etc. [41,45,55]. Only a small number of researchers have studied the effects of electrolytes and binders for TiO_2_-based anode in PIBs. It was known that traditional electrolyte systems of 0.8–1.0 M potassium hexafluorophosphate (KPF_6_) have been widely used. Developing a suitable electrolyte system for TiO_2_ anodes can effectively regulate its interfacial electrochemistry and provide stable solid electrolyte interphase (SEI) films.

**Figure 6:**
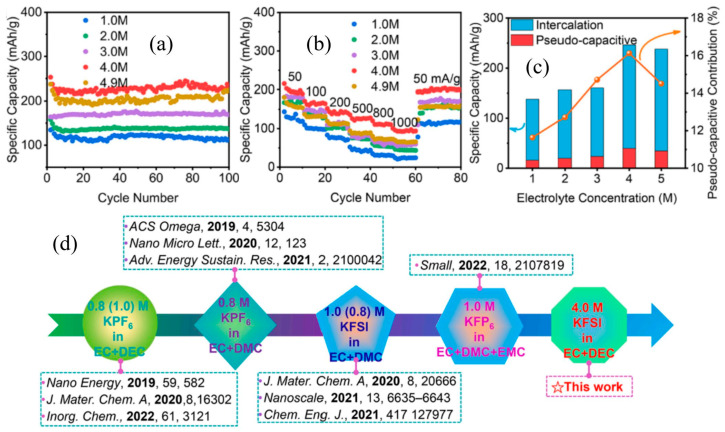
Cycling stability (**a**) and rate performance (**b**) of TiO_2_@C electrode in different concentrated electrolytes. (**c**) Corresponding capacity contributions of intercalation and pseudocapacitive reaction. (**d**) Electrolyte evolution for TiO_2_-based potassium storage systems. Reproduced with permission from Ref. [67] Copyright. 2022 Elsevier.

As shown in Figure 6, Lai et al. [67] investigated the influence of different potassium bis(fluorosulfonyl)imide (KFSI)-based electrolyte systems on the potassium storage performances of as-prepared TiO_2_@C anodes. Their research found that the optimized 4.0 M KFSI in a 1:1 (by volume) mixture of ethylene carbonate (EC) and diethyl carbonate (DEC) brings the best potassium storage performances. Typically, owing to the stable SEI film formed in this electrolyte system, the TiO_2_@C anode exhibits long cycling stability performance (shown in Figure 6a) and superior rate performance (shown in Figure 6b, 252 and 95 mA h g^−1^ at 50 and 1000 mA g^−1^, respectively). The capacity contribution shows an increasing tendency from 1.0 M to 4.0 M and then drops at 4.0 M (shown in Figure 6c). As shown in Figure 6d, the electrolyte system used in their work shows an obvious energy storage advantage in PIBs [67]. In addition, powder active materials are usually mixed into a slurry with a binder and then coated on a metal collector as test electrodes. Choosing different types of binders will have a certain impact on potassium storage performances for TiO_2_-based anodes. Yang et al. [68] investigated the effects of polyvinylidene difluoride (PVDF) and sodium carboxymethyl cellulose (CMC) used as a binder for TiO_2_ anodes. Their research results indicate that utilizing a CMC binder shows better potassium storage performances than that of a PVDF binder.

### 2.5. The Role as a Modifier

Intercalation-type TiO_2_-based anodes exhibit excellent energy storage performances in PIBs, owing to their intrinsic structural advantages, excellent electrochemical stability and mechanical strength, as well as flexible and facile preparation methods. Researchers can not only focus on exploring various modification and optimization methods but also skillfully utilize the potassium storage advantages and physical characteristics of TiO_2_ to modify other potassium storage anodes. Zhou et al. [69] prepared yolk-shell Bi_2_O_3_@TiO_2_ submicrospheres (y-Bi_2_O_3_@TiO_2_), obtaining enhanced potassium storage performances. As Bi_2_O_3_ is similar to other conversion-type anodes, it will undergo drastic volume expansion during the potassium storage process, leading to the pulverization and cracking of electrode materials. In this yolk-shell electrode structure, the introduction of an amorphous TiO_2_ shell can effectively alleviate the volume expansion effect caused by the K-ion insertion/extraction process. Thus, as for the application in PIBs, it delivers a superior cycling performance (216.8 mA h g^−1^ at 500 mA g^−1^ over 500 cycles), as well as a remarkable rate performance with a high specific capacity of 383.5 and 134.1 mA h g^−1^ at 0.1 and 2 A g^−1^, respectively [69].

As we know, the electrode/electrolyte interface can be regulated by coating the surface with some oxides. Zhao et al. [70] studied the impact of ultrathin TiO_2_ coating on SnS/rGO anodes in PIBs. An atomic layer deposition (ALD) system was used to prepare a TiO_2_ film. The research results indicate that charge transfer capability and K-ion diffusion kinetics can be effectively enhanced by the potassiated K*_x_*TiO_2_ coating layer derived from the TiO_2_ film. Moreover, Park et al. [71] used the layered-structured a-MoO_3_ anode as an example to investigate the effects of thin-film TiO_2_ ALD coating. Remarkable strain reduction can be observed, leading to enhanced cycling performance in aqueous PIBs. Typically, a TiO_2_ ALD layer 10 nm thick can reduce the lattice deformation by 68.2% and obtain better capacity retention 2.5 times bigger than that of a pristine electrode after 20 cycles. In addition, TiO_2_ can not only serve as a film-coating layer but also as a core structure. Xiong et al. [72] prepared a composite electrode of TiO_2_ core coated by amorphous MoS_3_ nanosheets wrapped by a carbon layer. Owing to the synergistic effects of TiO_2_, MoS_3_ and the carbon layer, as for the application in PIBs, it shows a high specific capacity of 463, 398, 333, 268, 189 and 104 mA h g^−1^ at the current density of 0.1, 0.2, 0.5, 1, 2 and 5 A g^−1^, respectively.

In addition, although various modification methods can effectively improve the potassium storage performances of TiO_2_, its inherent low theoretical specific capacity still limits its application. Based on the inherent structural advantages of TiO_2_, exploring how to leverage its energy storage advantages and compensate for its low specific capacity disadvantage is an important research topic. An effective solution is to composite with a high-specific-capacity anode, such as a conversion-type or alloy-type anode. The application of synergistic effects has been reported to significantly improve the energy storage performance of electrodes [73]. As we know, alloy-type phosphorus-based anodes have a high theoretical specific capacity of 865 mA h g^−1^ for PIBs [74]. However, their large volume expansion limits their application in PIBs. Feng et al. [74] prepared TiO_2_-red phosphorus/C nanofibers (TiO_2_-RP/CN) using electrospinning and annealing techniques. The composite electrode combines the merits of P with high capacity and TiO_2_ with excellent cycling stability. As a result, the TiO_2_-RP/CN electrode displays a higher specific capacity of 257.8 mA h g^−1^ than that of the TiO_2_/CN electrode (193.2 mA h g^−1^) after 500 cycles at the current density of 0.05 A g^−1^ for the application in PIBs. Its rate performance is also better than that of the TiO_2_/CN electrode. The enhanced potassium storage performance can be attributed to the synergistic effects of the TiO_2_ and P anode. Wang et al. [75] prepared a core-shell Bi@Void@TiO_2_ heterostructure for carbon nanofibers. Bi has a high theoretical specific capacity of 386 mA h g^−1^. Their research results indicate that the TiO_2_ shell can prevent carbon skeleton collapse and inhibit the agglomeration phenomenon of Bi during the charging/discharging process. As for the application in PIBs, this composite electrode shows a high specific capacity of 388.8 mA h g^−1^ at the current density of 0.05 A g^−1^, as well as a high capacity retention of 85.4% at 2 A g^−1^ after 3000 cycles. Table 1 shows the energy storage performances of the reported TiO_2_-based anodes for application in PIBs. It is obvious that TiO_2_-based anodes exhibit excellent potassium storage performances.

## 3. Other Ti-Based Oxide Anodes

Besides the extensively studied TiO_2_, other Ti-based oxide anodes such as the K-Ti-O family, Na_2_Ti_3_O_7_, Li_4_Ti_5_O_12_, etc., are of great interest for applications in PIBs. The K-Ti-O family with the general formula of K_2_Ti_n_O_2n+1_ has intrinsic potassium storage structural advantages, which can provide efficient K-ion insertion/extraction sites. In the K_2_Ti_n_O_2n+1_ family, several compounds, like K_2_Ti_4_O_9_, K_2_Ti_6_O_13_ and K_2_Ti_8_O_17_, have shown excellent potassium storage potential in PIBs.

### 3.1. K_2_Ti_6_O_13_ Anode

K_2_Ti_6_O_13_ has a monoclinic crystal phase with a C2/m space group. It matches the Joint Committee for Powder Diffraction Studies (JCPDS) reference card no. 40-0403 [76]. The structure of K_2_Ti_6_O_13_ is similar to the A_2_Ti_6_O_13_ (A = H, Li, Na) compounds. It has a tunnel structure, where edge-shared TiO_6_ octahedra are connected by corners. Such a structure could provide stable and effective tunnels for K-ion storage and transport [76,77]. Jiao et al. [78] revealed the solid-solution storage mechanism of the K_2_Ti_6_O_13_ anode. In addition, Sun et al. [77] prepared K_2_Ti_6_O_13_/carbon core-shell nanorods to study their PIB performances. The existent long-axis (010) and short-axis (001) crystal orientations bring fast K-ion diffusion behavior. This composite electrode shows superior rate performance (122.5, 104.3, 92.3, 78.6 and 65.1 mA h g^−1^ at 20, 50, 100, 200 and 500 mA g^−1^, respectively), as well as good cycling performance. Based on the potassium storage advantage of K_2_Ti_6_O_13_, Zhang et al. [76] designed and studied a novel potassium-ion hybrid capacitor using K_2_Ti_6_O_13_ microscaffolds as the anode, which shows an outstanding cycling performance over 5000 cycles.

**Figure 7:**
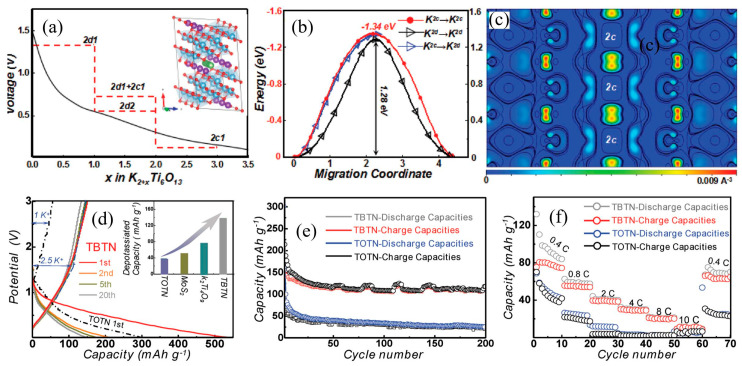
(**a**) Calculated voltage profiles for K_2_Ti_6_O_13_ upon potassiation. (**b**) Energy barrier profiles of K-ion diffusion. (**c**) Differential charge density of K_2_Ti_6_O_13_. Charge–discharge profiles (**d**), cycling performance (**e**) and rate performance (**f**) of TBTN and TOTN electrodes. Reproduced with permission from Ref. [79]. Copyright 2018 Wiley.

As shown in Figure 7, Wang et al. [79] studied the potassiation modeling of K_2_Ti_6_O_13_ using density functional theory (DFT) calculations. Three voltage plateaus can be observed according to the calculated reaction pathway (shown in Figure 7a). The K-ion migration energy of the 2d site to the 2d site is near that of the 2d site to the 2c site (shown in Figure 7b). The results in Figure 7c indicate the different bonds of inserted K-ions at 2d and 2c sites with adjacent O atoms. Moreover, they also investigated its size-dependent solid-solution behavior through K-ion insertion into K_2_Ti_6_O_13_ nanowires with different diameters. Their research results indicate that the K_2_Ti_6_O_13_ nanowires with an average diameter of about 5.5 nm (in short as TBTN) exhibit better rate and cycling performances than those of TOTN (diameter 38 nm) in PIBs (shown in Figure 7d–f) [79]. As we can see, reducing the particle size could be helpful in improving the energy storage performances of K_2_Ti_6_O_13_ anodes.

### 3.2. K_2_Ti_4_O_9_ and K_2_Ti_8_O_17_ Anodes

K_2_Ti_4_O_9_ is known to have a monoclinic structure with a C2/m space group. It has a layered lattice structure, in which K-ions occupy the interlayer spacing. Owing to the advantage of providing potassium storage sites in the interlayer spacing, it exhibits potential applications in PIBs. The potassium insertion/extraction reaction mechanism of K_2_Ti_4_O_9_ can be expressed by the following equation: K_2_Ti_4_O_9_ + 2K^+^ + 2e^−^ ⇔ K_4_Ti_4_O_9_. Munichandraiah et al. [80] synthesized K_2_Ti_4_O_9_ using the solid-state method and then investigated its energy storage performances in PIBs for the first time. It shows a specific capacity of 80 and 97 mA h g^−1^ at the rate of 0.8 C and 0.2 C, respectively. Moreover, with the aid of the alkalization process of Ti_3_C_2_ MXene, Wu et al. [81] prepared ultrathin K_2_Ti_4_O_9_ nanoribbons (M-KTO). Owing to the large interlayer spacing of 0.93 nm and open macroporous network of M-KTO, it shows a specific capacity of 151 and 88 mA h g^−1^ at the current density of 50 and 300 mA g^−1^, respectively. In addition, Ti_3_C_2_T*_x_*@K_2_Ti_4_O_9_ was prepared by Yan et al. [82] using an oxidation and alkalization method. The energy storage advantages of MXene are discussed by us in Section 2.3. K_2_Ti_4_O_9_ is considered a “zero strain” energy storage material [83]. As a result, the effective recombination of K_2_Ti_4_O_9_ and MXene brings a large specific surface area and shortened ion diffusion distance, as well as a stable energy storage structure. As for the application in PIBs, this composite electrode displays a high specific capacity of 164.3 mA h g^−1^ at 100 mA g^−1^ and excellent cycling performance (120.1 mA h g^−1^ at 200 mA g^−1^ after 2000 cycles) [82].

As for K_2_Ti_8_O_17_, if all Ti^4+^ in it is reduced to Ti^3+^, the theoretical specific capacity of K_2_Ti_8_O_17_ as the anode in PIBs is 308 mA h g^−1^ [84]. It has a monoclinic structure similar to K_2_Ti_4_O_9_. The edge-/corner-sharing TiO_6_ octahedrons create its layered structure, which furnishes suitable interlayer spacing and open channels for K-ion transport and storage. Xu et al. [84] systematically studied the potassium storage performances of the K_2_Ti_8_O_17_ anode. The potential potassium storage performance was obtained with a specific capacity of 44.2 mA h g^−1^ at the current density of 500 mA g^−1^.

### 3.3. Na_2_Ti_3_O_7_ and Li_4_Ti_5_O_12_ Anodes, etc.

**Figure 8:**
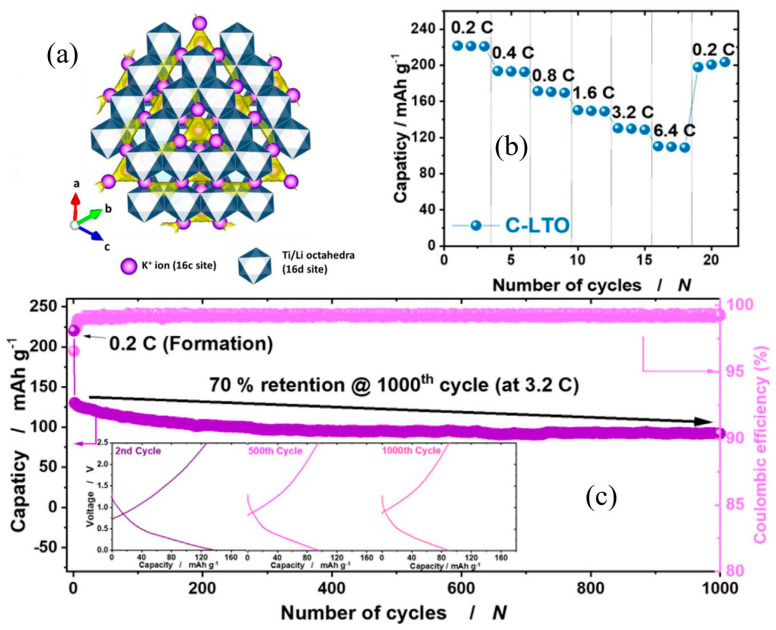
(**a**) Schematic illustration of possible sites of K-ion intercalation in rock-salt-type K_6_LiTi_5_O_12_. Rate performance (**b**) and cycling stability (**c**) of pre-potassiated C-LTO electrode. Reproduced with permission from Ref. [91]. Copyright 2021 Elsevier.

As we know, Na_2_Ti_3_O_7_ has been widely studied in NIBs owing to its inherent layered structure and open framework [36,85,86]. It also has potential advantages in the application of PIBs. Guo et al. [87] certified the potential potassium storage performances of the Na_2_Ti_3_O_7_-based anode. A specific capacity of 107.8 mA h g^−1^ can be obtained at the current density of 100 mA g^−1^ after 20 cycles. It also has a high capacity retention of 82.5% after 1555 cycles. However, poor electric conductivity and sluggish K-ion kinetics of Na_2_Ti_3_O_7_ remain the main challenges limiting its application in PIBs. Similar to Na_2_Ti_3_O_7_, Li_4_Ti_5_O_12_ has been extensively studied in LIBs and NIBs owing to its spinel structure, which has large space to accommodate lithium and sodium ions [35,88,89,90]. However, there are few research reports on its application in PIBs. In 2021, Myung et al. [91] first investigated the potential potassium storage application of carbon-modified Li_4_Ti_5_O_12_ (C-LTO). K-ions can be expected to occupy the vacant octahedral site (shown in Figure 8a). Their experimental results have certified that larger K-ions could be inserted into the 16c site of the cubic spinel structure. It induces a reversible biphasic transition to form a cubic rock salt structure: 2Li_4_Ti_5_O_12_ + 6K^+^ + 6e^−^ ⇔ Li_7_Ti_5_O_12_ + K_6_LiTi_5_O_12_, as accompanied by Ti^4+^/Ti^3+^ redox. Moreover, with the aid of improved electrical conductivity derived from carbon coating, this pre-potassiated C-LTO electrode exhibits a high specific capacity of 221 and 110 mA h g^−1^ at the rate of 0.2C (34 mA g^−1^) and 6.4C (1.08 A g^−1^), respectively (shown in Figure 8b). As shown in Figure 8c, it also shows a high reversible specific capacity of 130 mA h g^−1^ even at a high rate of 3.2C for 1000 cycles, corresponding to a 70% capacity retention [91]. In addition, ultrathin titanate nanosheets/graphene films prepared by Qiao et al. [92] show superior potassium storage performances. Wang et al. [93] synthesized carbon-coated K_2_Ti_2_O_5_ microspheres, which show enhanced K-ion intercalation pseudocapacitive behavior, accompanied by fast potassium storage and long cycling performances.

## 4. Conclusions and Perspectives

Ti-based oxides including TiO_2_, K_2_Ti_6_O_13_, K_2_Ti_4_O_9_, K_2_Ti_8_O_17_, Na_2_Ti_3_O_7_, Li_4_Ti_5_O_12_, etc., have attracted considerable attention for application in PIBs owing to their advantages of an intrinsic energy storage structure. Most of them have various merits such as excellent chemical and thermal stability, abundant reserves, non-toxic and environmentally friendly properties and facile preparation methods. However, their large bandgap indicates poor electrical conductivity, coupled with poor ion mobility, which seriously limits the application prospects of Ti-based oxides as anodes in PIBs. Although various modification methods, including structural regulation, carbon modification, element doping, etc., can effectively improve their potassium storage performances, there are still some related issues and problems that need to be addressed and solved when facing future applications.

Firstly, there is limited research on related theoretical calculations of Ti-based oxide anodes in PIBs. In order to better understand and optimize the potassium storage characteristics of Ti-based oxide anodes, it is necessary to conduct in-depth theoretical calculation research, including the crystal structure, spatial steric resistance, migration path and barrier range when K-ions are embedded into active materials. In addition, some key issues related to the potassium storage process, such as storage mechanism, dendrite growth and side reaction issues, also need to be focused on.

Secondly, due to the larger size of K^+^ (0.138 nm) compared to Na^+^ (0.102 nm) and Li^+^ (0.076 nm), the larger radius of K-ions will lead to slow ion diffusion kinetics during the insertion/extraction process, significantly affecting their potassium storage performances. This is also an inherent bottleneck barrier problem that must be faced by all Ti-based oxide anodes. Developing anode materials with fast potassium storage kinetics is still a key research direction for Ti-based oxide anodes. In addition, exploring effective modification methods to enhance their electrical conductivity will be an important research direction that should be dedicated to in the future. Among these modification methods, structural optimization, carbon modification, element doping, etc., will still be the mainstream strategies.

Finally, research on the effects of electrolytes and binders on potassium storage for Ti-based oxide anodes should be strengthened in the future, as well as the role of TiO_2_ as a modifier. As for the practical application issue of Ti-based oxides in PIBs, TiO_2_ shows great potential for practical application. However, most TiO_2_-based anodes with excellent potassium storage performances are prepared through complex and cumbersome modification methods. This is not conducive to future practical applications. Therefore, as for the future modification process, it is necessary to take both the potassium storage performances and the simplicity of the modification method into account. In addition, the current research system is mostly based on half-cells and lacks relevant full-cell research. Thus, in order to accelerate practical applications, more efforts should be devoted to the investigation of full cells for Ti-based oxide anodes in PIBs.

## Figures and Tables

**Table 1:** Energy storage performances of the reported TiO_2_-based anodes for application in PIBs.

Materials	Potassium Storage Performances	References
TiO_2_/NC-HN	277.2, 244.7, 210.2, 185.5, 165.7, 126.9 and 79.1 mA h g^−1^ at 0.1, 0.2, 0.5, 1.0, 2.0, 5.0 and 10.0 A g^−1^, respectively.	[45]
HeTiO_2_eC MTs	208.5, 180.1, 114.6 and 97.3 mA h g^−1^ at 0.2, 0.4, 1 and 2 A g^−1^, respectively.	[40]
TO/C	176.2, 155.0, 131.4, 115.8, 93.1, 62.3, 36.3 and 27.9 mA h g^−1^ at 0.1, 0.2, 0.5, 1, 2, 5, 10 and 20 C rate, respectively.	[47]
G-TiO_2_ NTs	271.6, 258.7, 217.3, 189.3, 166.8, 133.4 and 129.2 mA h g^−1^ at 0.05, 0.1, 0.2, 0.5, 1, 2 and 5 A g^−1^, respectively.	[41]
TiO_2_-5-Ta/CNF	187.9, 170.9, 154.1, 140.6, 137.6, 126.3, 118.5 and 102.8 mA h g^−1^ at 0.1, 0.2, 0.4, 0.8, 1, 2, 3 and 5 A g^−1^, respectively.	[55]
TiO_2_-RP/CN	248.9, 221.8, 199.8, 178.8, 158.7 and 151.4 mA h g^−1^ at 0.05, 0.1, 0.2, 0.4, 0.8 and 1 A g^−1^, respectively	[74]
TiSe_2_/TiO_2_/C	245, 191, 142, 105, 68 and 43 mA h g^−1^ at 0.1, 0.2, 0.5, 1, 2 and 5 A g^−1^, respectively	[62]
Ti_3_C_2_/TiO_2_/rGO	592.5, 413.9, 349.1, 293.5 and 221.7 mA h g^−1^ at 0.1, 0.2, 0.3, 0.5, 1.0 A g^−1^, respectively	[60]
TiO_2_/RGO	354.3, 282.2, 220.9, 151.7 and 107.1 mA h g^−1^ at 0.05, 0.1, 0.2, 0.5 and 1 A g^−1^, respectively.	[61]
MOF-NOC@L-TiO_2_	555, 355, 253, 179, 130, 87 and 73 mA h g^−1^ at 0.05, 0.1, 0.2, 0.5, 1, 2 and 3 A g^−1^, respectively	[63]
y-Bi_2_O_3_@TiO_2_	373.9, 347.7, 299.6, 252.8, 207.7 and 139.1 mA h g^−1^ at 0.1, 0.2, 0.3, 0.5, 1.0 and 2.0 A g^−1^, respectively	[69]
Bi@Void@TiO_2_ CNF	388.8, 301.9, 281.4, 230.5, 191.5, 159.7, 90 and 64.9 mA h g^−1^ at 0.05, 0.1, 0.2, 0.5, 1, 2, 5 and 10 A g^−1^, respectively	[75]
TiO_2_@A-MoS_3_ @NC	463, 398, 333, 268, 189 and 104 mA h g^−1^ at 0.1, 0.2, 0.5, 1, 2 and 5 A g^−1^, respectively.	[72]

## Data Availability

Data sharing is not applicable to this article.

## References

[1] Lu L., Han X., Li J., Hua J., Ouyang M. (2013). A review on the key issues for lithium-ion battery management in electric vehicles. J. Power Sources.

[2] Wu F., Maier J., Yu Y. (2020). Guidelines and trends for next-generation rechargeable lithium and lithium-ion batteries. Chem. Soc. Rev..

[3] Li M., Lu J., Chen Z., Amine K. (2018). 30 Years of Lithium-Ion Batteries. Adv. Mater..

[4] Li J., Fleetwood J., Hawley W.B., Kays W. (2022). From Materials to Cell: State-of-the-Art and Prospective Technologies for Lithium-Ion Battery Electrode Processing. Chem. Rev..

[5] Harper G., Sommerville R., Kendrick E., Driscoll L., Slater P., Stolkin R., Walton A., Christensen P., Heidrich O., Lambert S. (2019). Recycling lithium-ion batteries from electric vehicles. Nature.

[6] Neumann J., Petranikova M., Meeus M., Gamarra J.D., Younesi R., Winter M., Nowak S. (2022). Recycling of Lithium-Ion Batteries-Current State of the Art, Circular Economy, and Next Generation Recycling. Adv. Energy Mater..

[7] Yabuuchi N., Kubota K., Dahbi M., Komaba S. (2014). Research Development on Sodium-Ion Batteries. Chem. Rev..

[8] Rajagopalan R., Tang Y., Ji X., Jia C., Wang H. (2020). Advancements and Challenges in Potassium Ion Batteries: A Comprehensive Review. Adv. Funct. Mater..

[9] Hwang J.-Y., Myung S.-T., Sun Y.-K. (2017). Sodium-ion batteries: Present and future. Chem. Soc. Rev..

[10] Hosaka T., Kubota K., Hameed A.S., Komaba S. (2020). Research Development on K-Ion Batteries. Chem. Rev..

[11] Hou H., Qiu X., Wei W., Zhang Y., Ji X. (2017). Carbon Anode Materials for Advanced Sodium-Ion Batteries. Adv. Energy Mater..

[12] Wu X., Chen Y., Xing Z., Lam C.W.K., Pang S.-S., Zhang W., Ju Z. (2019). Advanced Carbon-Based Anodes for Potassium-Ion Batteries. Adv. Energy Mater..

[13] Min X., Xiao J., Fang M., Wang W., Zhao Y., Liu Y., Abdelkader A.M., Xi K., Kumar R.V., Huang Z. (2021). Potassium-ion batteries: Outlook on present and future technologies. Energ. Environ. Sci..

[14] Zhang W., Liu Y., Guo Z. (2019). Approaching high-performance potassium-ion batteries via advanced design strategies and engineering. Sci. Adv..

[15] Xu Y.-S., Duan S.-Y., Sun Y.-G., Bin D.-S., Tao X.-S., Zhang D., Liu Y., Cao A.-M., Wan L.-J. (2019). Recent developments in electrode materials for potassium-ion batteries. J. Mater. Chem. A.

[16] Wang N., Chu C., Xu X., Du Y., Yang J., Bai Z., Dou S. (2018). Comprehensive New Insights and Perspectives into Ti-Based Anodes for Next-Generation Alkaline Metal (Na+, K+) Ion Batteries. Adv. Energy Mater..

[17] Xu Y., Zhang J., Li D. (2020). Recent Developments in Alloying-type Anode Materials for Potassium-Ion Batteries. Chem. Asian J..

[18] Sha M., Liu L., Zhao H., Lei Y. (2020). Anode materials for potassium-ion batteries: Current status and prospects. Carbon. Energy.

[19] Zhang C., Zhao H., Lei Y. (2020). Recent Research Progress of Anode Materials for Potassium-ion Batteries. Energy Environ. Mater..

[20] Liu S., Kang L., Henzie J., Zhang J., Ha J., Amin M.A., Hossain M.S.A., Jun S.C., Yamauchi Y. (2021). Recent Advances and Perspectives of Battery-Type Anode Materials for Potassium Ion Storage. ACS Nano.

[21] Gu Y., Pei Y.R., Zhao M., Yang C.C., Jiang Q. (2022). Sn-, Sb- and Bi-Based Anodes for Potassium Ion Battery. Chem. Rec..

[22] Song K., Liu C., Mi L., Chou S., Chen W., Shen C. (2021). Recent Progress on the Alloy-Based Anode for Sodium-Ion Batteries and Potassium-Ion Batteries. Small.

[23] Lei K.-X., Wang J., Chen C., Li S.-Y., Wang S.-W., Zheng S.-J., Li F.-J. (2020). Recent progresses on alloy-based anodes for potassium-ion batteries. Rare Met..

[24] Lu S., Wu H., Xu S., Wang Y., Zhao J., Li Y., Abdelkader A.M., Li J., Wang W., Xi K. (2021). Iron Selenide Microcapsules as Universal Conversion-Typed Anodes for Alkali Metal-Ion Batteries. Small.

[25] Sha D., You Y., Hu R., Cao X., Wei Y., Zhang H., Pan L., Sun Z. (2023). Comprehensively Understanding the Role of Anion Vacancies on K-Ion Storage: A Case Study of Se-Vacancy-Engineered VSe_2_. Adv. Mater..

[26] Deng Q., Fu Y., Zhu C., Yu Y. (2019). Niobium-Based Oxides Toward Advanced Electrochemical Energy Storage: Recent Advances and Challenges. Small.

[27] Jian Z., Hwang S., Li Z., Hernandez A.S., Wang X., Xing Z., Su D., Ji X. (2017). Hard-Soft Composite Carbon as a Long-Cycling and High-Rate Anode for Potassium-Ion Batteries. Adv. Funct. Mater..

[28] Xu Y., Zhang C., Zhou M., Fu Q., Zhao C., Wu M., Lei Y. (2018). Highly nitrogen doped carbon nanofibers with superior rate capability and cyclability for potassium ion batteries. Nat. Commun..

[29] Chen G., Chen J., Zhao S., He G., Miller T.S. (2023). Pseudohexagonal Nb_2_O_5_ Anodes for Fast-Charging Potassium-Ion Batteries. ACS Appl. Mater. Interfaces.

[30] Li X., Li J., Ma L., Yu C., Ji Z., Pan L., Mai W. (2022). Graphite Anode for Potassium Ion batteries: Current Status and Perspective. Energy Environ. Mater..

[31] Zhao Z., Cheng J., Li K., Li C., Zhang S., Pei X., Niu Z., Liu Z., Fu Y., Li D. (2021). Heterojunction interfacial promotion of fast and prolonged alkali-ion storage of urchin-like Nb_2_O_5_@C nanospheres. J. Mater. Chem. A.

[32] Guo S., Yi J., Sun Y., Zhou H. (2016). Recent advances in titanium-based electrode materials for stationary sodium-ion batteries. Energ. Environ. Sci..

[33] Lou S., Zhao Y., Wang J., Yin G., Du C., Sun X. (2019). Ti-Based Oxide Anode Materials for Advanced Electrochemical Energy Storage: Lithium/Sodium Ion Batteries and Hybrid Pseudocapacitors. Small.

[34] Vazquez-Santos M.B., Tartaj P., Morales E., Manuel Amarilla J. (2018). TiO_2_ Nanostructures as Anode Materials for Li/Na-Ion Batteries. Chem. Rec..

[35] Yuan T., Tan Z., Ma C., Yang J., Ma Z.-F., Zheng S. (2017). Challenges of Spinel Li_4_Ti_5_O_12_ for Lithium-Ion Battery Industrial Applications. Adv. Energy Mater..

[36] Xie F., Zhang L., Su D., Jaroniec M., Qiao S.-Z. (2017). Na_2_Ti_3_O_7_@N-Doped Carbon Hollow Spheres for Sodium-Ion Batteries with Excellent Rate Performance. Adv. Mater..

[37] Li R., Lin C., Wang N., Luo L., Chen Y., Li J., Guo Z. (2018). Advanced composites of complex Ti-based oxides as anode materials for lithium-ion batteries. Adv. Compos. Hybrid. Ma..

[38] Paul S., Rahman M.A., Bin Sharif S., Kim J.-H., Siddiqui S.-E.T., Hossain M.A.M. (2022). TiO_2_ as an Anode of High-Performance Lithium-Ion Batteries: A Comprehensive Review towards Practical Application. Nanomaterials.

[39] Liang S., Wang X., Qi R., Cheng Y.-J., Xia Y., Mueller-Buschbaum P., Hu X. (2022). Bronze-Phase TiO_2_ as Anode Materials in Lithium and Sodium-Ion Batteries. Adv. Funct. Mater..

[40] Li Y., Yang C., Zheng F., Pan Q., Liu Y., Wang G., Liu T., Hu J., Liu M. (2019). Design of TiO_2_eC hierarchical tubular heterostructures for high performance potassium ion batteries. Nano Energy.

[41] Cai J., Cai R., Sun Z., Wang X., Wei N., Xu F., Shao Y., Gao P., Dou S., Sun J. (2020). Confining TiO_2_ Nanotubes in PECVD-Enabled Graphene Capsules Toward Ultrafast K-Ion Storage: In Situ TEM/XRD Study and DFT Analysis. Nano-Micro Lett..

[42] Reeves K.G., Ma J., Fukunishi M., Salanne M., Komaba S., Dambournet D. (2018). Insights into Li+, Na+, and K+ Intercalation in Lepidocrocite-Type Layered TiO_2_ Structures. ACS Appl. Energy Mater..

[43] Deng Q., Yao L. (2022). Self-Standing Soft Carbon-Coated MoS2 Nanofiber Film Anode for Superior Potassium Storage. Coatings.

[44] Zang Q., Zhang Q., Luo Z., Liao L., Ouyang X., Xie S. (2022). Construction of high conductivity carbon-coated MoS_2_ on porous carbon nanofibers for synergistic potassium storage. J. Power Sources.

[45] Wang G., Li Y., Liu Y., Jiao S., Peng B., Li J., Yu L., Zhang G. (2021). Nest-like TiO_2_-nitrogen-doped-carbon hybrid nanostructures as superior host for potassium-ion hybrid capacitors. Chem. Eng. J..

[46] Luo R., Ma Y., Qu W., Qian J., Li L., Wu F., Chen R. (2020). High Pseudocapacitance Boosts Ultrafast, High-Capacity Sodium Storage of 3D Graphene Foam-Encapsulated TiO_2_ Architecture. ACS Appl. Mater. Interfaces.

[47] Ling L., Wang X., Zhou M., Wu K., Lin C., Younus H.A., Zhang M., Zhang S., Cheng F., Zhang Y. (2022). Carbon-Coated Flower-Like TiO_2_ Nanosphere as an Ultrastable Anode Material for Potassium-Ion Batteries: Structure Design and Mechanism Study. ACS Appl. Energy Mater..

[48] Li W., Gao N., Cheng S., Wu J., Chen Q. (2022). Electrochemical Performance of Sandwich-like Structured TiO_2_/graphene Composite as Anode for Potassium-ion Batteries. Int. J. Electrochem. Sci..

[49] Dubal D.P., Schneemann A., Ranc V., Kment S., Tomanec O., Petr M., Kmentova H., Otyepka M., Zboril R., Fischer R.A. (2021). Ultrafine TiO_2_ Nanoparticle Supported Nitrogen-Rich Graphitic Porous Carbon as an Efficient Anode Material for Potassium-Ion Batteries. Adv. Energy Sustain. Res..

[50] Liao J., Hu Q., Mu J., Chen F., He X., Chen F., Wen Z., Chen C. (2020). Introducing a conductive pillar: A polyaniline intercalated layered titanate for high-rate and ultra-stable sodium and potassium ion storage. Chem. Commun..

[51] Cao J., Zhong J., Xu H., Li S., Deng H., Wang T., Fan L., Wang X., Wang L., Zhu J. (2022). N/S co-doped carbon nanosheet bundles as high-capacity anode for potassium-ion battery. Nano Res..

[52] Hwang K., Sohn H., Yoon S. (2018). Mesostructured niobium-doped titanium oxide-carbon (Nb-TiO_2_-C) composite as an anode for high-performance lithium-ion batteries. J. Power Sources.

[53] Zhang W., Luo N., Huang S., Wu N.-L., Wei M. (2019). Sulfur-Doped Anatase TiO_2_ as an Anode for High-Performance Sodium-Ion Batteries. ACS Appl. Energy Mater..

[54] Fan M., Lin Z., Zhang P., Ma X., Wu K., Liu M., Xiong X. (2021). Synergistic Effect of Nitrogen and Sulfur Dual-Doping Endows TiO_2_ with Exceptional Sodium Storage Performance. Adv. Energy Mater..

[55] Su D., Liu L., Liu Z., Dai J., Wen J., Yang M., Jamil S., Deng H., Cao G., Wang X. (2020). Electrospun Ta-doped TiO_2_/C nanofibers as a high-capacity and long-cycling anode material for Li-ion and K-ion batteries. J. Mater. Chem. A.

[56] Cui J., Yin P., Xu A., Jin B., Li Z., Shao M. (2022). Fluorine enhanced nucleophilicity of TiO_2_ nanorod arrays: A general approach for dendrite-free anodes towards high-performance metal batteries. Nano Energy.

[57] Xiong D., Li X., Bai Z., Lu S. (2018). Recent Advances in Layered Ti_3_C_2_T*_x_* MXene for Electrochemical Energy Storage. Small.

[58] Pang J., Mendes R.G., Bachmatiuk A., Zhao L., Ta H.Q., Gemming T., Liu H., Liu Z., Rummeli M.H. (2019). Applications of 2D MXenes in energy conversion and storage systems. Chem. Soc. Rev..

[59] Bashir T., Zhou S., Yang S., Ismail S.A., Ali T., Wang H., Zhao J., Gao L. (2023). Progress in 3D-MXene Electrodes for Lithium/Sodium/Potassium/Magnesium/Zinc/Aluminum-Ion Batteries. Electrochem. Energy R..

[60] Wu S., Feng Y., Wu K., Jiang W., Xue Z., Xiong D., Chen L., Feng Z., Wen K., Li Z. (2023). Mxene Ti_3_C_2_ generated TiO_2_ nanoparticles in situ and uniformly embedded in rGO sheets as high stable anodes for potassium ion batteries. J. Alloys Compd..

[61] Fang Y., Hu R., Liu B., Zhang Y., Zhu K., Yan J., Ye K., Cheng K., Wang G., Cao D. (2019). MXene-derived TiO_2_/reduced graphene oxide composite with an enhanced capacitive capacity for Li-ion and K-ion batteries. J. Mater. Chem. A.

[62] Qi F., Shao L., Lu X., Liu G., Shi X., Sun Z. (2022). MXene-derived TiSe_2_/TiO_2_/C heterostructured hexagonal prisms as high rate anodes for Na-ion and K-ion batteries. Appl. Surf. Sci..

[63] Zhang R., Tian Y., Otitoju T., Feng Z., Wang Y., Sun T. (2023). Sand-Fixation Model for Interface Engineering of Layered Titania and N/O-Doped Carbon Composites to Enhance Potassium/Sodium Storage. Small.

[64] Xu Y., Ding T., Sun D., Ji X., Zhou X. (2023). Recent Advances in Electrolytes for Potassium-Ion Batteries. Adv. Funct. Mater..

[65] Xing J., Bliznakov S., Bonville L., Oljaca M., Maric R. (2022). A Review of Nonaqueous Electrolytes, Binders, and Separators for Lithium-Ion Batteries. Electrochem. Energy R..

[66] Zhang Q., Wang Z., Zhang S., Zhou T., Mao J., Guo Z. (2018). Cathode Materials for Potassium-Ion Batteries: Current Status and Perspective. Electrochem. Energy R..

[67] Zheng J., Hu C., Nie L., Zang S., Chen H., Chen N., Ma M., Lai Q. (2023). Electrolyte manipulation enhanced pseudo-capacitive K-storage for TiO_2_ anode. Appl. Surf. Sci..

[68] Wang C., Su L., Wang N., Lv D., Wang D., Yang J., Qian Y. (2022). Unravelling binder chemistry in sodium/potassium ion batteries for superior electrochemical performances. J. Mater. Chem. A.

[69] Xu Y., Zhang H., Ding T., Tian R., Sun D., Wang M.-S., Zhou X. (2022). Synthesis of yolk-shell Bi_2_O_3_@TiO_2_ submicrospheres with enhanced potassium storage. Sci. China Chem..

[70] Wen S., Gu X., Ding X., Zhang L., Dai P., Li L., Liu D., Zhang W., Zhao X., Guo Z. (2022). Constructing ultrastable electrode/electrolyte interface for rapid potassium ion storage capability via salt chemistry and interfacial engineering. Nano Res..

[71] Schuppert N.D., Mukherjee S., Bates A.M., Son E.-J., Choi M.J., Park S. (2016). Ex-situ X-ray diffraction analysis of electrode strain at TiO2 atomic layer deposition/alpha-MoO_3_ interface in a novel aqueous potassium ion battery. J. Power Sources.

[72] Huang M., Xi B., Mi L., Zhang Z., Chen W., Feng J., Xiong S. (2022). Rationally Designed Three-Layered TiO_2_@amorphous MoS3@Carbon Hierarchical Microspheres for Efficient Potassium Storage. Small.

[73] Deng Q., Chen F., Liu S., Bayaguud A., Feng Y., Zhang Z., Fu Y., Yu Y., Zhu C. (2020). Advantageous Functional Integration of Adsorption-Intercalation-Conversion Hybrid Mechanisms in 3D Flexible Nb_2_O_5_@Hard Carbon@MoS_2_@Soft Carbon Fiber Paper Anodes for Ultrafast and Super-Stable Sodium Storage. Adv. Funct. Mater..

[74] Su D., Dai J., Yang M., Wen J., Yang J., Liu W., Hu H., Liu L., Feng Y. (2021). Red phosphorus embedded in TiO_2_/C nanofibers to enhance the potassium-ion storage performance. Nanoscale.

[75] Gao Z., Han L., Gao H., Chen J., Sun Z., Zhu C., Zhang Y., Shi J., Chen S., Wang H. (2022). Coupling core-shell Bi@Void@TiO_2_ heterostructures into carbon nanofibers for achieving fast potassium storage and long cycling stability. J. Mater. Chem. A.

[76] Dong S., Li Z., Xing Z., Wu X., Ji X., Zhang X. (2018). Novel Potassium-Ion Hybrid Capacitor Based on an Anode of K_2_Ti_6_O_13_ Microscaffolds. ACS Appl. Mater. Interfaces.

[77] Liu C., Wang H., Zhang S., Han M., Cao Y., Liu S., Yang Z., Chen A., Sun J. (2020). K_2_Ti_6_O_13_/carbon core-shell nanorods as a superior anode material for high-rate potassium-ion batteries. Nanoscale.

[78] Cao K., Liu H., Li W., Xu C., Han Q., Zhang Z., Jiao L. (2019). K_2_Ti_6_O_13_ nanorods for potassium-ion battery anodes. J. Electroanal. Chem..

[79] Xu S.-M., Ding Y.-C., Liu X., Zhang Q., Wang K.-X., Chen J.-S. (2018). Boosting Potassium Storage Capacity Based on Stress-Induced Size-Dependent Solid-Solution Behavior. Adv. Energy Mater..

[80] Kishore B., Venkatesh G., Munichandraiah N. (2016). K_2_Ti_4_O_9_: A Promising Anode Material for Potassium Ion Batteries. J. Electrochem. Soc..

[81] Dong Y., Wu Z.-S., Zheng S., Wang X., Qin J., Wang S., Shi X., Bao X. (2017). Ti_3_C_2_ MXene-Derived Sodium/Potassium Titanate Nanoribbons for High-Performance Sodium/Potassium Ion Batteries with Enhanced Capacities. ACS Nano.

[82] Ma Z., Li Q., Pang H., Yu Z., Yan D. (2022). Ti_3_C_2_T*_x_*@K_2_Ti_4_O_9_ composite materials by controlled oxidation and alkalization strategy for potassium ion batteries. Ceram. Int..

[83] Zheng X., Li P., Zhu H., Zhao G., Rui K., Shu J., Xu X., Wang X., Sun W., Dou S.X. (2020). Understanding the structural and chemical evolution of layered potassium titanates for sodium ion batteries. Energy Storage Mater..

[84] Han J., Xu M., Niu Y., Li G.-N., Wang M., Zhang Y., Jia M., Li C.M. (2016). Exploration of K_2_Ti_8_O_17_ as an anode material for potassium-ion batteries. Chem. Commun..

[85] Pan H., Lu X., Yu X., Hu Y.-S., Li H., Yang X.-Q., Chen L. (2013). Sodium Storage and Transport Properties in Layered Na_2_Ti_3_O_7_ for Room-Temperature Sodium-Ion Batteries. Adv. Energy Mater..

[86] Fu S., Ni J., Xu Y., Zhang Q., Li L. (2016). Hydrogenation Driven Conductive Na_2_Ti_3_O_7_ Nanoarrays as Robust Binder-Free Anodes for Sodium-Ion Batteries. Nano Lett..

[87] Li P., Wang W., Gong S., Lv F., Huang H., Luo M., Yang Y., Yang C., Zhou J., Qian C. (2018). Hydrogenated Na_2_Ti_3_O_7_ Epitaxially Grown on Flexible N-Doped Carbon Sponge for Potassium-Ion Batteries. ACS Appl. Mater. Interfaces.

[88] Yi T.-F., Yang S.-Y., Xie Y. (2015). Recent advances of Li_4_Ti_5_O_12_ as a promising next generation anode material for high power lithium-ion batteries. J. Mater. Chem. A.

[89] Li J., Chang X., Huang T., Wang B., Zheng H., Luo Q., Peng D.-L., Wei Q. (2023). Surface-controlled sodium-ion storage mechanism of Li_4_Ti_5_O_12_ anode. Energy Storage Mater..

[90] Natarajan S., Subramanyan K., Aravindan V. (2019). Focus on Spinel Li_4_Ti_5_O_12_ as Insertion Type Anode for High-Performance Na-Ion Batteries. Small.

[91] Kim H.J., Jo J.H., Choi J.U., Voronina N., Ahn D., Jeon T.-Y., Yashiro H., Aniskevich Y., Ragoisha G., Streltsov E. (2021). Long Life Anode Material for Potassium Ion Batteries with High-Rate Potassium Storage. Energy Storage Mater..

[92] Zeng C., Xie F., Yang X., Jaroniec M., Zhang L., Qiao S.-Z. (2018). Ultrathin Titanate Nanosheets/Graphene Films Derived from Confined Transformation for Excellent Na/K Ion Storage. Angew. Chem. Int. Edit..

[93] Zhao S., Dong L., Sun B., Yan K., Zhang J., Wan S., He F., Munroe P., Notten P.H.L., Wang G. (2020). K_2_Ti_2_O_5_@C Microspheres with Enhanced K^+^ Intercalation Pseudocapacitance Ensuring Fast Potassium Storage and Long-Term Cycling Stability. Small.

